# Oxycodone versus fentanyl for intravenous patient-controlled analgesia after laparoscopic supracervical hysterectomy: A prospective, randomized, double-blind study

**DOI:** 10.1097/MD.0000000000006286

**Published:** 2017-03-10

**Authors:** Nan Seol Kim, Jeong Seok Lee, Su Yeon Park, Aeli Ryu, Hea Rim Chun, Ho Soon Chung, Kyou Sik Kang, Jin Hun Chung, Kyung Taek Jung, Seong Taek Mun

**Affiliations:** a Department of Anesthesiology and Pain Medicine, Soonchunhyang University Hospital Cheonan, 23–20, Byeongmyeong-dong, Dongnam-gu, Cheonan, Chungcheongnam-do; b Department of anesthesiology and pain medicine, Soonchunhyang University Bucheon Hospital, Soonchunhyang University College of Medicine, Bucheon-si, Gyeonggi-do; c Department of Biostatistics, Soonchunhyang University Seoul Hospital, Soonchunhyang University College of Medicine, Seoul; d Department of obstetrics and gynecology, Soonchunhyang University Cheonan Hospital, Soonchunhyang University College of Medicine, Cheonan-si, Chungcheongnam-do, Republic of Korea.

**Keywords:** Fentanyl, oxycodone, postoperative pain

## Abstract

**Background:**

Oxycodone, a semisynthetic thebaine derivative opioid, is widely used for the relief of moderate to severe pain. The aim of this study was to compare the efficacy and side effects of oxycodone and fentanyl in the management of postoperative pain by intravenous patient-controlled analgesia (IV-PCA) in patients who underwent laparoscopic supracervical hysterectomy (LSH).

**Methods:**

The 127 patients were randomized to postoperative pain treatment with either oxycodone (n = 64, group O) or fentanyl group (n = 63, group F). Patients received 7.5 mg oxycodone or 100 μg fentanyl with 30-mg ketorolac at the end of anesthesia followed by IV-PCA (potency ratio 75:1) for 48 hours postoperatively. A blinded observer assessed postoperative pain based on the numerical rating scale (NRS), infused PCA dose, patient satisfaction, sedation level, and side effects.

**Results:**

Accumulated IV-PCA consumption in group O was less (63.5 ± 23.9 mL) than in group F (85.3 ± 2.41 mL) during the first 48 hours postoperatively (*P* = 0.012). The NRS score of group O was significantly lower than that of group F at 4 and 8 hours postoperatively (*P* < .001); however, the incidence of postoperative nausea and vomiting (PONV), dizziness, and drowsiness was significantly higher in group O than in group F. Patient satisfaction was lower in group O than in group F during the 48 hours after surgery (*P* < 0.001).

**Conclusions:**

Oxycodone IV-PCA (potency ratio 1:75) provided superior analgesia to fentanyl IV-PCA after LSH; however, the higher incidence of side effects, including PONV, dizziness, and drowsiness, suggests that the doses used in this study were not equipotent.

## 1 Introduction

In response to developments in surgical and anesthetic techniques, postoperative morbidity and mortality have rapidly decreased, allowing anesthetists to focus on specific issues related to an operation. Eberhart et al^[1]^ reported that postoperative nausea and vomiting (PONV), as well as pain, are major concerns of surgical patients. Accordingly, methods to relieve both have been examined in several studies.

Inadequate postoperative pain control may result in harmful physiological and psychological reactions that lead to postoperative complications. In addition, the hindered recovery may delay the resumption of normal daily activities, thus decreasing the patient's degree of satisfaction, especially if chronic pain develops.^[2]^ Among the various methods of controlling postoperative pain, intravenous patient-controlled analgesia (IV-PCA) has been used extensively, based on its improved pain control and minimal side effects.^[3]^

Dihydrohydroxycodeinone (oxycodone) is a semisynthetic opioid derived from thebaine, extracted from the opium poppy; as a μ-opioid receptor agonist, it is used for the control of moderate to severe pain.^[4]^ Fentanyl is an opioid analgesic most commonly used in Korea in postoperative IV-PCA. Intravenous oxycodone was recently approved by Korea's Ministry of Food and Drug Safety (MFDS) for IV-PCA and released onto the market in 2013. Consequently, clinical experience regarding the efficacy of oxycodone in IV-PCA is limited.^[5]^

In the implementation of IV-PCA, the choice of an appropriate analgesic and control of the appropriate dose and lockout interval are critical to the reduction of side effects and the achievement of effective analgesia. The administration of oxycodone at too high a dose to patients sensitive to opioids may not only cause minor side effects, such as PONV, but also respiratory depression, bradycardia, apnea, hypotension, circulatory collapse, respiratory arrest, and death.^[5]^ Therefore, the safe, recommended doses of fentanyl and oxycodone, the 2 opioid analgesics frequently used in IV-PCA, must be carefully determined, taking into account the direct conversion factor. Previous reports have shown that the dose conversion ratio from morphine to oxycodone and from fentanyl to morphine is 1:1^[6]^ and 1:100,^[7,8]^ respectively. The fentanyl–oxycodone dose conversion ratio was estimated indirectly as 1:100, whereas studies performed based on the estimated ratio showed that the appropriate dose conversion ratio was 1:75.^[9,10]^]

In the present study, using a fentanyl–oxycodone dose conversion ratio of 1:75 on the basis of previous reports, postoperative pain measured with the numerical rating scale (NRS), infused PCA dose, side effects, and degree of satisfaction were compared in laparoscopic supracervical hysterectomy (LSH) patients who were administered IV-PCA fentanyl or oxycodone for 48 hours postoperatively.

## 2 Materials and methods

This study was approved by the institutional review board at Soonchunhyang University Cheonan Hospital, Korea. The 130 adult patients were between the ages of 18 and 65 years, with American Society of Anesthesiologists (ASA) physical status of 1 or 2, and had requested postoperative IV-PCA for LSH scheduled to be performed at our hospital. Patients with a history of bleeding tendency, hepatitis, or renal failure; those with habitual sedative or other drug use; those with psychiatric disease; and those who were inappropriate candidates for IV-PCA were excluded from the study.

On the day before surgery, patients were informed of the purpose of the study, the PCA method, NRS, and the possible side effects of either form of IV-PCA. All patients included in the study provided written informed consent, after which they were randomly allocated to either the fentanyl (fentanyl citrate; Guju, Seoul, Korea) group (group F, n = 65) or the oxycodone (OxyNorm; Mundipharma, Seoul, Korea) group (group O, n = 65). Group allocation was performed by a blinded observer according to a computer-generated simple randomization code.

On the morning before the operation, vascular access was secured using an 18-G needle. The patients were transported to the operation room without preanesthetic medication. Upon their arrival, they were connected to an electrocardiograph, a noninvasive blood pressure monitor, and a pulse oximeter. Anesthesia was induced by the intravenous injection of 2 mg propofol/kg, and 0.8 mg rocuronium bromide/kg (Esmeron, Organon, Netherlands). Following endotracheal intubation, oxygen and air were supplied at a fraction of inspired oxygen (FiO_2_) of 0.4 together with desflurane (end-tidal concentration of 6.0–7.0 vol %), maintaining an end-tidal carbon dioxide level of 35 to 40 mmHg. To ensure an appropriate anesthetic depth, the desflurane concentration was controlled so that blood pressure and heart rate values were held within 20% of the preoperative measurements; 0.5 to 1 μg fentanyl/kg was used for analgesia as necessary. After completion of the operation, desflurane and air were discontinued. Pyridostigmine and glycopyrrolate were injected to reverse the muscle relaxation. Patients were extubated after verifying a sufficient recovery of consciousness and spontaneous respiration. The IV-PCA device (Ambix Anapa; IWha-Fresenius Kabi, Seoul, Korea) was then connected. Patients in group F were injected with a loading dose of 100 μg of fentanyl and 30 mg of ketorolac, and those in group O with 7.5 mg of oxycodone and 30 mg of ketorolac. All patients were administered 0.3 mg of ramosetron to prevent PONV, the most common side effect of IV-PCA. For group F patients, 700 μg of fentanyl, 150 mg of ketorolac, and 0.6 mg of ramosetron were mixed with saline to a total volume of 100 mL; continuous infusion was set to 14 μg/h, a bolus dose of 0.5 mL, and a lockout interval of 15 minutes. For group O, based on a fentanyl–oxycodone dose conversion ratio of 1:75, 52.5 mg of oxycodone, 150 mg of ketorolac, and 0.6 mg of ramosetron were mixed with saline to a total volume of 100 mL; continuous infusion was set to 1050 μg/h, a bolus dose of 0.5 mL, and a 15-minute lockout interval. These settings were maintained for 48 hours. The study was double-blinded regarding the type of IV-PCA. At 0.5, 4, 8, 24, and 48 hours after LSH, the quantity of analgesics used by each patient, the administration of additional analgesics, pain intensity, the sedation level, and side effects, including PONV, were assessed and the data were recorded by a single-blinded observer.

The intensity of pain was assessed with the NRS, in which a score of “0” corresponds to “no pain” and “100” to “worst pain imaginable.” In patients with a NRS score between 30 and 50, representing moderate pain, if the established dose of analgesics had been injected and the patient requested further pain control, an additional intravenous injection of 30 mg of ketolorac was administered. In patients with a NRS score ≥50, representing high-intensity pain, the request for further pain control was met by an additional intravenous injection of 1 μg fentanyl per kg.

The sedation level was assessed using the modified observer's assessment of alertness/sedation (MOAA/S) scale (0, does not respond to noxious stimuli; 1, does not respond to mild prodding or shaking; 2, responds only after mild prodding or shaking; 3, responds only after name is called loudly or repeatedly; 4, lethargic response to name spoken in normal tone; 5, responds readily to name spoken in normal tone). Respiratory depression was defined as a respiratory rate of <8 breaths/min. In patients with a MOAA/S scale score ≤1, or in those with respiratory depression, PCA was immediately stopped and 0.01 mg naloxone/kg was intravenously injected. Other side effects (PONV, dizziness, pruritus, and headache) were also monitored. Patients with severe PONV received an additional intravenous injection of 10 mg of metoclopramide. Those with pruritus were intravenously injected with 25 mg of pheniramine.

Patient satisfaction, graded as very satisfied, satisfied, neutral, and dissatisfied, was measured at 8 and 48 hours postoperatively.

### 2.1 Statistical analysis

The effective number of patients was determined with reference to a previous study.^[11]^ The primary endpoint was thus determined as the NRS score at 30 minutes postoperatively. The clinically significant effect size was an average primary endpoint difference between the 2 groups of 7.5, with a standard deviation of 15.28. Calculation of the sample size showed that at a significance level (α) of 0.05 (1-sided) and power (1–β) of 0.8, 52 patients were required for each group. With an expected dropout rate of 10%, the final estimated number of participants in each group was 65.

The statistical analysis was performed using SPSS software for Windows (version 18, SPSS Inc, Chicago, IL). Measurement data are expressed as the mean and standard deviation. Demographic data were analyzed using Pearson *χ*^2^ test or Student *t* test. The generalized estimating equation method was used to compare IV-PCA quantity and pain level between the 2 groups postoperatively for 48 hours, both at rest and during coughing. A *P* value <0.001 was considered to be statistically significant. The incidence of side effects and the degree of patient satisfaction were compared in groups F and O using Pearson *χ*^2^ test or Fisher exact test. A *P* value <0.05 was considered to be statistically significant.

## 3 Results

The 130 patients enrolled in the study were randomized with respect to treatment, with 65 patients allocated to each of the 2 groups. Three patients withdrew from the study: 1 patient in group O did not meet the inclusion criteria and was therefore excluded, and 1 patient each in groups O and F discontinued treatment because of PONV. Therefore, 127 patients completed the study: 64 in group F and 63 in group O.

There were no statistically significant differences between the 2 groups with respect to age, height, weight, duration of surgery or anesthesia, ASA physical status, motion sickness, or smoking history (Table 1).

**Table 1:**
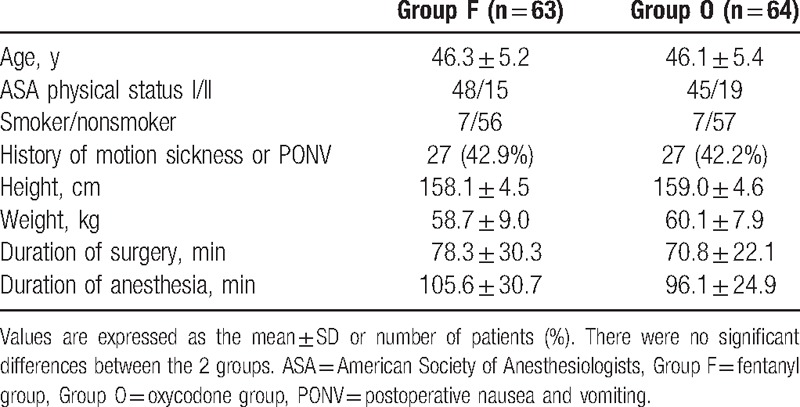
Demographic data and anesthesia characteristics of the patients.

**Figure 1:**
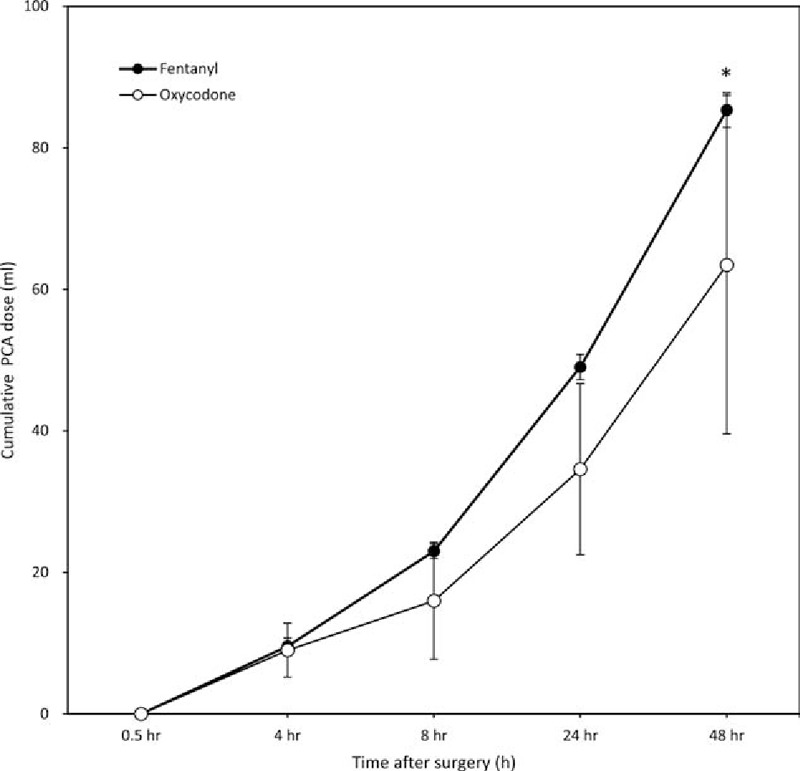
Accumulated fentanyl and oxycodone consumption (mL) 0.5 to 48 hours postoperatively (mean ± standard deviation). ^∗^*P* < 0.001 for an interaction effect between group and time. The *P* value was calculated using the generalized estimating equation method. PCA = patient-controlled analgesia.

The 2 groups did not significantly differ with respect to infused PCA dose at 30 minutes or 4, 8, 24, or 48 hours postoperatively; however, the amount of PCA during the first 48 hours after surgery was significantly less in group O than in group F (Fig. 1).

**Figure 2:**
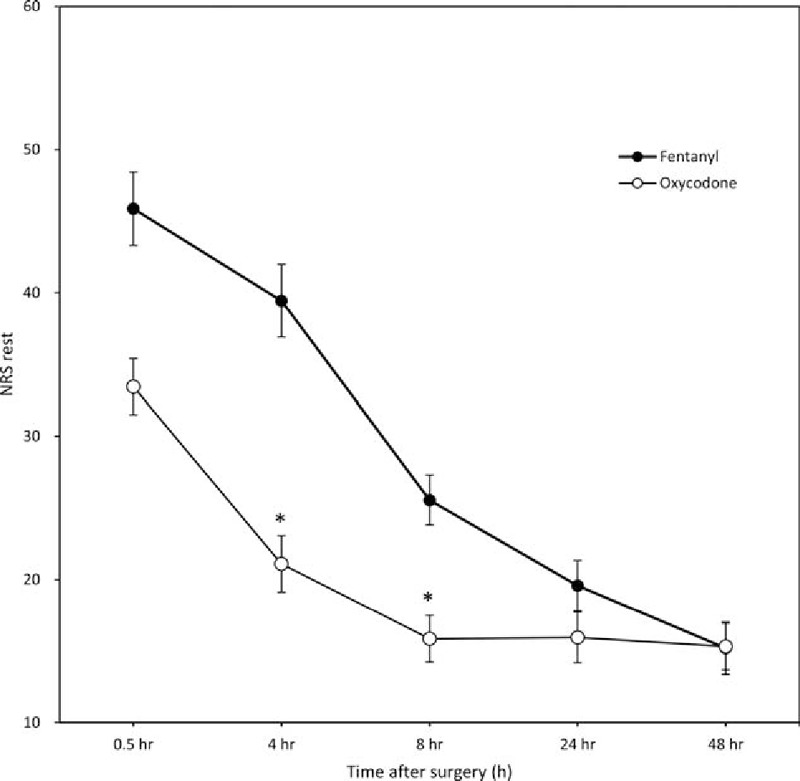
NRS at rest 0.5 to 48 hours postoperatively (mean ± standard deviation). The possible range is between 0 and 100 mm.^∗^*P* < 0.001 for an interaction effect between group and time. The *P* value was calculated using the generalized estimating equation method. NRS = numeric rating scale.

**Figure 3:**
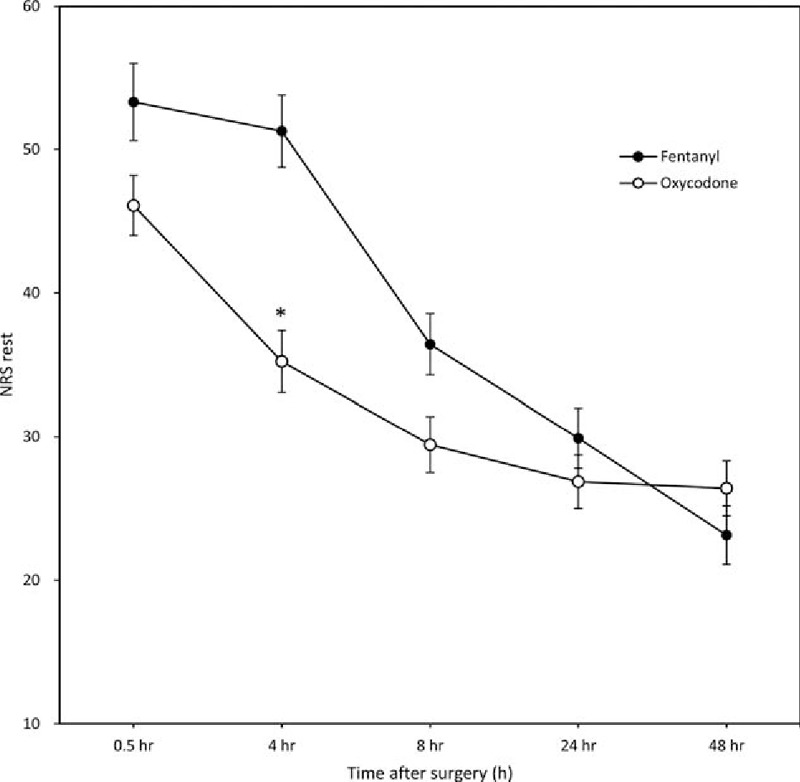
NRS during coughing 0.5 to 48 hours postoperatively (mean ± standard deviation). The possible range is between 0 and 100 mm. ^∗^*P* < 0.001 for an interaction effect between group and time. The *P* value was calculated using the generalized estimating equation method. NRS = numeric rating scale.

The difference between the 2 groups in the NRS at rest was not significantly different at 0.5, 24, or 48 hours postoperatively, but at 4 and 8 hours, it was significantly lower in group O than in group F (*P* < 0.001) (Fig. 2). The NRS on coughing was significantly lower in group O than in group F only at 4 hours postoperatively (*P* < 0.001) (Fig. 3).

The nausea level at 4, 8, 24, and 48 hours, but not at 0.5 hour, was significantly higher in group O than in group F. The incidence of postoperative vomiting was significantly higher in group O only at 8 hours postoperatively (Table 2), as was the administration of additional analgesics (*P* < 0.05) (Table 3). In contrast, the administration of additional antiemetic drugs was significantly more frequent in group F at 8 h postoperatively (Table 3) whereas, overall, dizziness and drowsiness occurred significantly more often in group O (Table 4). Respiratory depression was not observed in either of the 2 groups, nor were there significant differences in their sedation scores.

**Table 2:**
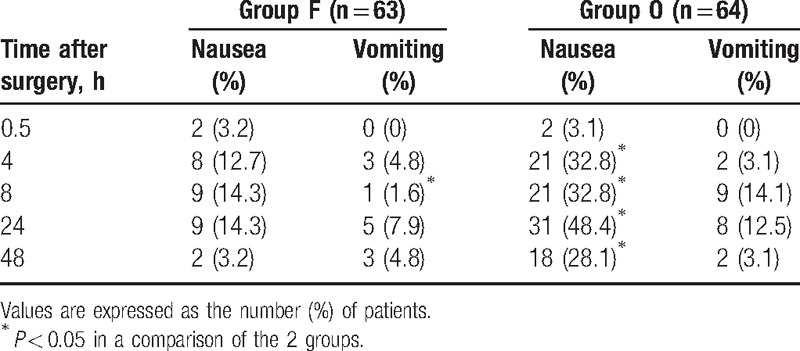
Incidence (%) of postoperative nausea and vomiting.

**Table 3:**
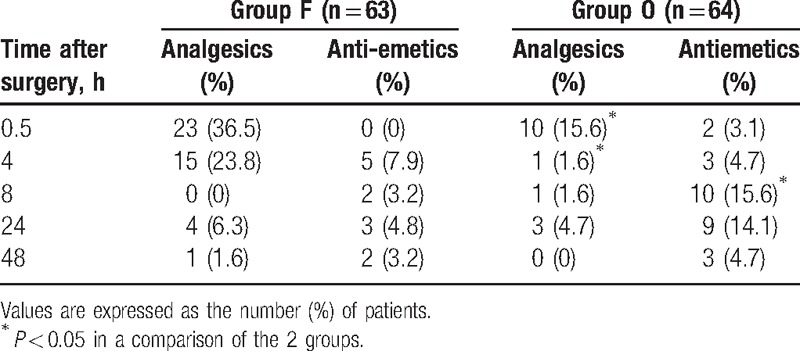
Use of rescue analgesics and antiemetics.

**Table 4:**
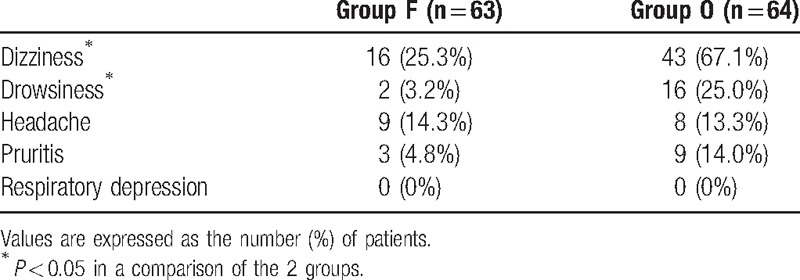
Incidence (%) of adverse events.

Postoperative patient satisfaction also did not significantly differ between the 2 groups at 8 hours postoperatively; however, at 48 hours, it was significantly higher in group F than in group O (Table 5).

**Table 5:**
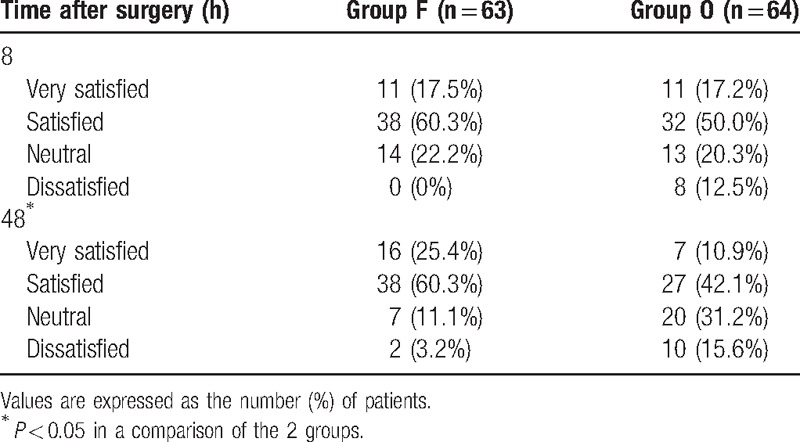
Patient satisfaction at 8 and 48 hours postoperatively.

## 4 Discussion

Generally, laparoscopic surgery offers several advantages over laparotomy, including less pain, faster recovery, and an improved cosmetic effect.^[12]^ However, patients frequently experience moderate pain even after “minor” surgery because analgesics are used postoperatively at doses smaller than those actually required.^[13]^ The establishment of an appropriate IV-PCA dose is important for the postoperative management also of patients undergoing minor surgery. This study compared the analgesic effect, PCA quantity, side effects, and degree of satisfaction in the use of IV-PCA in patients who underwent LSH, a relatively minor operation. Fentanyl is commonly used in IV-PCA in our hospital, whereas oxycodone is a newly released intravenous analgesic with a shorter history of use in IV-PCA. The fentanyl–oxycodone dose conversion ratio used in our study was 1:75.

The opioids widely administered for IV-PCA are full μ-receptor-binding agonists, with no analgesic ceiling in pain control. However, a clinical ceiling set to prevent side effects, such as sedation and, particularly, respiratory depression, may prevent the use of an appropriate pain control dose.^[3]^ Ketorolac, when used in combination with opioids, has an opioid-sparing effect, enhancing the analgesic effects of opioids while reducing their side effects.^[14]^ Therefore, we included ketorolac in the IV-PCA to reduce both the side effects of opioids and the pain caused by motion.^[2,15]^

Fentanyl, which is a 4-amilidopiperidine compound, has extremely high lipid solubility and a rapid onset of action. Its analgesic effect occurs within 30 seconds after injection and reaches a peak in 5 minutes, reflecting its fast and extensive redistribution in the body. Thus, fentanyl has the properties of an ideal IV-PCA drug, as well as the advantage of not producing active metabolites that may cause respiratory depression.^[3,7]^

Oxycodone, a newly released intravenous analgesic, is a semisynthetic opioid that was developed in Germany in 1916 in an attempt to improve opioids.^[4]^ It is structurally analogous to morphine, which is a global reference standard for opioids, and has a 1:1 analgesic strength in postoperative pain involving somatic and visceral pain factors.^[6]^ Oxycodone is metabolized in the liver by cytochrome P450 enzymes. However, because it acts on μ-opioid receptors, oxycodone has the typical side effects of other opioids, including nausea, constipation, drowsiness, vomiting, pruritus, and dizziness.^[4]^

Koch et al^[10]^ compared the effect of intravenous fentanyl and oxycodone for postoperative pain control in the recovery room during the first 2 postoperative hours in patients who underwent laproscopic cholecystectomy. The total amount of fentanyl used was 200 μg and that of oxycodone 15 mg, consistent with a fentanyl-oxycodone dose conversion ratio of ∼1:75. Hwang et al^[9]^ also reported a fentanyl–oxycodone dose conversion ratio of ∼1:75 during the 48 hours after laproscopic cholecystectomy, which suggested that oxycodone is more potent than morphine. Based on these results, we anticipated that fentanyl and oxycodone would produce similar analgesic effects when used in IV-PCA at a ratio of 1:75; however, this was not the case. Although, as expected, the NRS score at 30 minute postoperatively was not significantly different between groups O and F, the use of rescue analgesics was significantly more frequent in the latter group (Table 3). Additionally, for acute postoperative pain 4 and 8 hours postoperatively, the analgesic effect of oxycodone was superior to that of fentanyl (Figs. 1 and 2), and PCA consumption at 48 hours was significantly greater in group F (85.3 ± 2.41 mL vs 63.5 ± 23.9 mL, *P* = 0.012). The difference between the results of this study and previous ones can be explained by the shorter monitoring times (2 hours postoperatively) or the absence of continuous infusion in IV-PCA administered for 48 hours. The difference in the pharmacokinetics of the 2 drugs should be taken into account when IV-PCA, including continuous infusion, is used for the continuous injection of drugs over several days, as in the present study. The analgesic onset time of oxycodone is 2 to 3 minutes, which is as short as that of fentanyl, whereas oxycodone has a slightly longer duration of action (t1/2: 4 hour 52 minutes vs 3 hours 39 minutes). However, owing to redistribution, the analgesic effect of fentanyl after a single intravenous injection is much shorter than that of oxycodone.^[10]^

Female sex, nonsmoking behavior, history of PONV, and the intraoperative or postoperative use of an opioid are high-risk factors for PONV, which is among the most common postoperative side effect. The incidence of PONV in one study was 21% with 1 risk factor, 39% with 3 risk factors, 61% with 3 risk factors, and 79% with 4 risk factors.^[16]^ Administration of a preventive antiemetic drug is recommended for patients at high risk. Because most patients in our study had at least 3 risk factors, ramosetron was included as a PONV preventive antiemetic. However, despite its administration in group O, the incidence of PONV was ≥30% beginning 4 hours postoperatively. This rate was higher than expected and higher than that of group F, which indicates that the administration of oxycodone was excessive and that the fentanyl–oxycodone dose conversion ratio of 1:75 requires adjustment.

Our survey of postoperative patient satisfaction showed that the 2 groups did not significantly differ at 8 hours postoperatively, whereas at 48 hours, patient satisfaction was significantly higher in group F than in group O (Table 5). With regard to acute postoperative pain within 8 hours postoperatively, the analgesic effect of oxycodone was superior to that of fentanyl such that patient satisfaction was not significantly different between the 2 groups, although side effects, including PONV, were frequently observed in group O. In contrast, at 48 hours postoperatively, whereas the pain score was similar between the 2 groups, patient satisfaction was lower in group O than in group F because of the high incidences of PONV, dizziness, and drowsiness in oxycodone-treated patients.

The lack of a significant difference in the sedation score between the 2 groups probably reflected the fact that the patients included in this study were healthy, ≤65 years of age, and had an ASA physical status of 1 or 2.

The present study also had several limitations. First, the continuous infusion rate, lockout interval, and bolus dose could not be controlled because an Anapa PCA pump was used during IV-PCA to investigate the analgesic quantity during the first 48 postoperative hours, which was the primary outcome. Hence, the continuous infusion rate was not reduced even in patients with a sufficiently low NRS or in whom side effects, such as nausea, were reported. This resulted in an unnecessarily high incidence of PONV. In patients with severe side effects, the IV-PCA was closed and subsequently reopened only if the side effect had abated or if the patient requested the IV-PCA again. Therefore, the equianalgesic dose between fentanyl and oxycodone could not be accurately calculated. Second, patient age is an important factor in the use of oxycodone, with patients older than 70 years having an average oxycodone exposure 40% to 80% higher than that of young adult patients.^[17]^ Because our study included only patients who were 65 years or younger, a further study of patients older than 70 years of age is required.

In conclusion, oxycodone, administered at an fentanyl–oxycodone dose conversion ratio of 1:75, may be an appropriate drug that can replace fentanyl in IV-PCA, based on our finding that oxycodone is much more effective than fentanyl in relieving acute postoperative pain within the first 8 hours postoperatively. However, owing to the high incidence of side effects, especially PONV, the oxycodone–fentanyl dose conversion ratio must be adjusted.
